# Developmental Switch in Neurovascular Coupling in the Immature Rodent Barrel Cortex

**DOI:** 10.1371/journal.pone.0080749

**Published:** 2013-11-05

**Authors:** Christoph M. Zehendner, Simeon Tsohataridis, Heiko J. Luhmann, Jenq-Wei Yang

**Affiliations:** Institute of Physiology, University Medical Center of the Johannes Gutenberg-University, Mainz, Germany; Biological Research Centre of the Hungarian Academy of Sciences, Hungary

## Abstract

Neurovascular coupling (NVC) in the adult central nervous system (CNS) is a mechanism that provides regions of the brain with more oxygen and glucose upon increased levels of neural activation. Hemodynamic changes that go along with neural activation evoke a blood oxygen level-dependent (BOLD) signal in functional magnetic resonance imaging (fMRI) that can be used to study brain activity non-invasively. A correct correlation of the BOLD signal to neural activity is pivotal to understand this signal in neuronal development, health and disease. However, the function of NVC during development is largely unknown. The rodent whisker-to-barrel cortex is an experimentally well established model to study neurovascular interdependences. Using extracellular multi-electrode recordings and laser-Doppler-flowmetry (LDF) we show in the murine barrel cortex of postnatal day 7 (P7) and P30 mice *in vivo* that NVC undergoes a physiological shift during the first month of life. In the mature CNS it is well accepted that cortical sensory processing results in a rise in regional cerebral blood flow (rCBF). We show in P7 animals that rCBF decreases during prolonged multi-whisker stimulation and goes along with multi unit activity (MUA) fatigue. In contrast at P30, MUA remains stable during repetitive stimulation and is associated with an increase in rCBF. Further we characterize in both age groups the responses in NVC to single sensory stimuli. We suggest that the observed shift in NVC is an important process in cortical development that may be of high relevance for the correct interpretation of brain activity e.g. in fMRI studies of the immature central nervous system (CNS).

## Introduction

Sensory information in the mammalian cortex is processed in highly organized neuronal networks such as cortical columns[1,2]. Cortical information processing is critically dependent on the ability of the neuronal networks to dynamically adapt towards changes in energy and oxygen demands which is ensured by NVC also known as functional hyperaemia[3,4]. In the adult CNS cortical sensory processing leads to an increase in rCBF in the activated sensory cortical areas and a decrease of local levels of deoxygenated hemoglobin resulting in a positive BOLD signal[5]. Therefore the BOLD signal indirectly provides information on neural activity in a non-invasive manner in fMRI recordings[6]. The physiological basis for the interpretation of the BOLD signal with regard to neural activity is based on NVC[4–6]. Perturbances of this neurovascular interplay occur in disorders of the central nervous system such as stroke, Alzheimer’s disease, vascular dementia and preterm brain injury[7–9]. Therefore it is important to study NVC in functional disorders of the CNS, e.g. by fMRI. A better understanding of the mechanisms underlying alterations of NVC in health and disease will help to develop novel strategies to treat failures in NVC with the goal to restore brain function[5]. 

NVC has been well characterized in the mature CNS[6], which is reflected by the wide use of functional brain imaging by fMRI[10,11]. However, previous studies have reported conflicting results on hemodynamic changes during neural activation in the developing brain in humans as well as in animal models[12–15]. Colonnese et al.[13] have detected exclusively positive BOLD signals (local increase of oxygenated hemoglobin, hyperemia) in the sensory cortex in a setting of electrical paw stimulation in rats. In contrast a recent study by Kozberg et al.[14] demonstrated that newborn rats display an inverted hemodynamic response (negative BOLD signal) compared with adults (positive BOLD signal, hyperemia) upon electrical hind paw activation[14]. The authors also noted that care should be taken when using electrical stimulation as a stimulation paradigm because it may induce perturbances in systemic blood pressure which may act as a confounder in the developing brain and may explain why some groups found positive BOLD signals upon electrical stimulation in immature rodents[14]. In humans Chiron and colleagues have shown that rCBF in the CNS changes with brain maturation[16]. It should be noted that clinical studies regarding NVC in the developing brain are often limited regarding the number of subjects studied, heterogeneity of patient cohorts, lack of follow ups or confounders e.g. diseases that go along with prematurity[14]. However these clinical and basic research data indicate that NVC undergoes significant changes during maturation (for detailed review also see Harris et al.[4]). These NVC changes may be highly dependent on the developmental stage of the CNS[4,16] and the experimental stimulation protocol used in order to evoke sensory processing[14,17]. 

The rodent whisker-to-barrel cortex is a well established sensory system in order to investigate neurovascular coupling[18–20] and has the following advantages as a model system:

• sensory stimulation can be induced in a non-painful physiological manner by whisker deflection[18,19,21,22]
• it is a well studied sensory system regarding anatomical representation of cortical columns as well as synaptic connectivity[1,2,23].

Despite these advantages as an experimental model system the question how neural processing and activity relates to changes in rCBF during distinct physiological sensory stimulation paradigms of the immature rodent whisker-to-barrel cortex has so far been neglected. Therefore the goals of the present study were to determine:

i) the neurovascular response to single stimulation of multiple whiskers in immature cortical columns of P7 mice compared with P30 animals;

ii) differences in NVC in the barrel cortex of P7 and P30 mice during prolonged (60 s) multi-whisker stimulation, which is a well established stimulation protocol to characterize neurovascular responses[18,19].

## Methods

### Ethics statement

All experiments were conducted in accordance with the national laws for the use of animals in research and approved by the local ethical committee of the University Medical Center Mainz and the local authorities “Landesuntersuchungsamt Rheinland-Pfalz” Aktenzeichen (protocol number): 23177-07/G 10-1-010 and G 12-1-070. All efforts were made to reduce the number of animals used and to minimize their suffering.

### General surgical preparation

**Figure 1:**
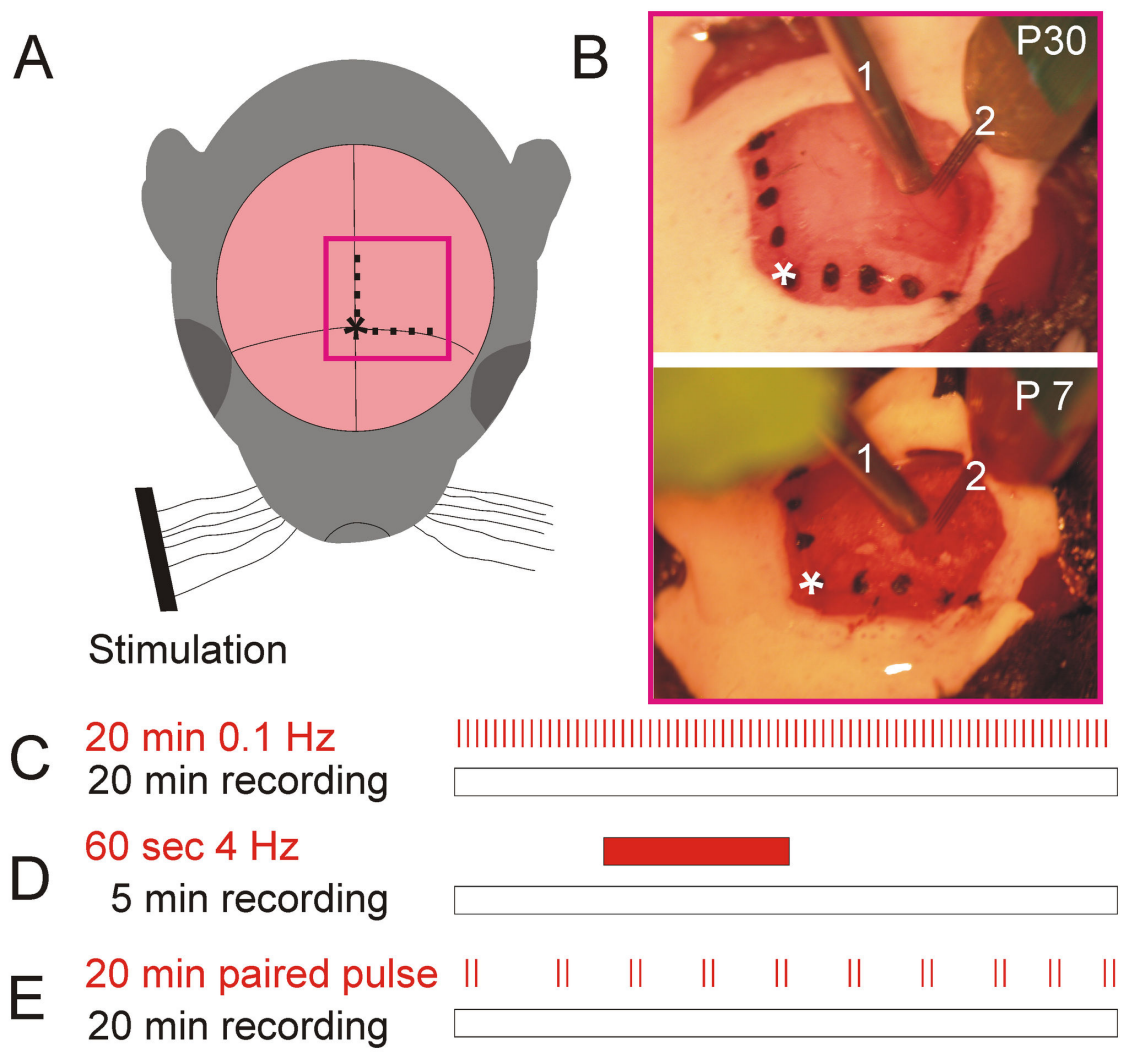
Experimental setup and stimulation protocols. For NVC analyses the bregma position (asterisk) and localization of the barrel cortex were identified. Barrel cortex was activated by mechanical stimulation of multiple whiskers (**A**). A craniotomy was performed and the LDF probe (1) was positioned 300 - 500 µm above the barrel cortex. A four-shank 16-channel electrode (2) was inserted into the barrel cortex at a depth of 200 - 300 µm (**B**). To detect neurovascular whisker-evoked cortical responses different stimulation protocols were used: 0.1 Hz stimulation for 20 minutes in order to detect NVC responses upon single multi-whisker stimulation (**C**); 4 Hz stimulation lasting 60 seconds during a total recording period of 5 minutes to investigate NVC in prolonged stimulation (**D**); Paired pulse stimulations at different frequencies for 20 minutes to elucidate neuronal inhibition at various time points after initial whisker stimulation (**E**).

In the current study we used urethane as anesthetic agent because urethane is widely used to investigate NVC due to its balanced effect on neurotransmitters[20]. Extracellular recordings were performed in the barrel cortex of two age groups (postnatal days (P) P7 and P27 to 34 C57/BL6 mice, both sexes were investigated). Mice at the age of P27-34 are referred to P30. Under initial intraperitoneal urethane anesthesia (1g/kg for P7 mice and 1.5 g/kg for P30 mice, Sigma-Aldrich), the head of the mouse was fixed and the bone except the dura mater above the barrel cortex was carefully removed (craniotomy coordinates P7: 2-3 mm lateral, 1-2 mm posterior bregma; coordinates P30: 2-4 mm lateral, 1-3 posterior bregma) similar as described elsewhere[21,22]. Animals were kept at a constant temperature of 37° C by placing them on a heating blanket. During recordings, additional urethane (10 to 25 % of the initial dose) was given when the mice showed any sign of distress. Using a piezoelectric sensor depth of anesthesia was controlled by monitoring breathing as described in detail before[24]. After 30-60 min recovery, a four-shank 16-channel electrode (125 µm horizontal shank distance and 50 µm vertical inter-electrode distance, 1-2 MΩ, NeuroNexus Technologies, Ann Arbor, MI) was inserted perpendicularly into cortical layer II/III and layer IV in a depth of 200 to 250 µm in P7 and 250 to 300 µm in P30 animals to obtain local field potential (LFP) recordings and multi-unit activity (MUA) from these layers. One hour after the electrode was inserted into the barrel cortex each single barrel-related column was identified by single whisker stimulation. Usually recordings were obtained from 1 to 3 barrels with this four-shank 16-channel electrode setting. The channel with the maximum evoked response was chosen for further analysis. After functional identification of the barrels one LDF probe (Oxyflo MNP100XP-3/15 Probe, Oxford Optronix) was positioned 300 - 500 µm above the barrel cortex closely to the four-shank 16-channel electrode (Figure **1**)

### Recordings and experimental protocol for neurovascular coupling in cortical columns

To depict processes of NVC in P7 and P30 mice, whiskers were cut to about 0.5 cm length and stimulated with a custom-made stimulator. The stimulator was composed of a miniature solenoid actuator that was driven by a transistor-transistor logic pulse (adapted from[22,25]). The stimulator was adjusted to deflect the whiskers near the tips of the whiskers (Figure **1 A**) which were cut to about 5 mm in order to reduce variances in extent of whisker deflections. Length of pulse duration was 10 ms. Both cortical LFP and MUA were recorded contralateral to sensory stimulation at a sampling rate of 20 kHz using a multi-channel extracellular amplifier and MC_RACK software (Multi Channel Systems, Reutlingen, Germany). The relative changes in rCBF were detected contralateral using LDF and were recorded with CED power 1401 as well as spike2 software (Cambridge Electronic Design, ENGLAND) at a sampling rate of 1 kHz (Figure **1 B**). Several different stimulation paradigms were used. To characterize the temporal relationship of neuronal and rCBF response multiple whiskers were stimulated at a frequency of 0.1 Hz for 20 minutes per animal (Figure **1 C**). One hundred responses per animal (n = 5 animals per age group) were averaged to minimize individual variation in sensory evoked multi-whisker responses. We have chosen a frequency of 0.1 Hz because at this frequency neuronal excitability at P7 reveals a stable level (see below). To mimic prolonged sensory processing whiskers were deflected for 60 s at 4 Hz and neurovascular responses were recorded for 5 minutes (n = 4 animals per group Figure **1 D**). Four Hz was found to give the best signal to noise ratio in our experimental setting. Eight to 11 measurements were performed per animal and were averaged to minimize individual discrepancies in the recordings. To evaluate neuronal excitability paired pulses at various frequencies (1, 0.5, 0.2, 0.1, 2 Hz) were applied and LFP as well as MUA were recorded for 20 minutes (Figure **1 E**). Paired pulses were separated from each other at an interval of 20 s. In paired pulse experiments a single whisker was stimulated and the neuronal recordings were obtained from the corresponding barrel. Throughout the experiments breathing rate was monitored with a piezoelectric element as previously described[24]. Body temperature was controlled using a thermometer and kept at 37°C using a heating pad.

**Figure 2:**
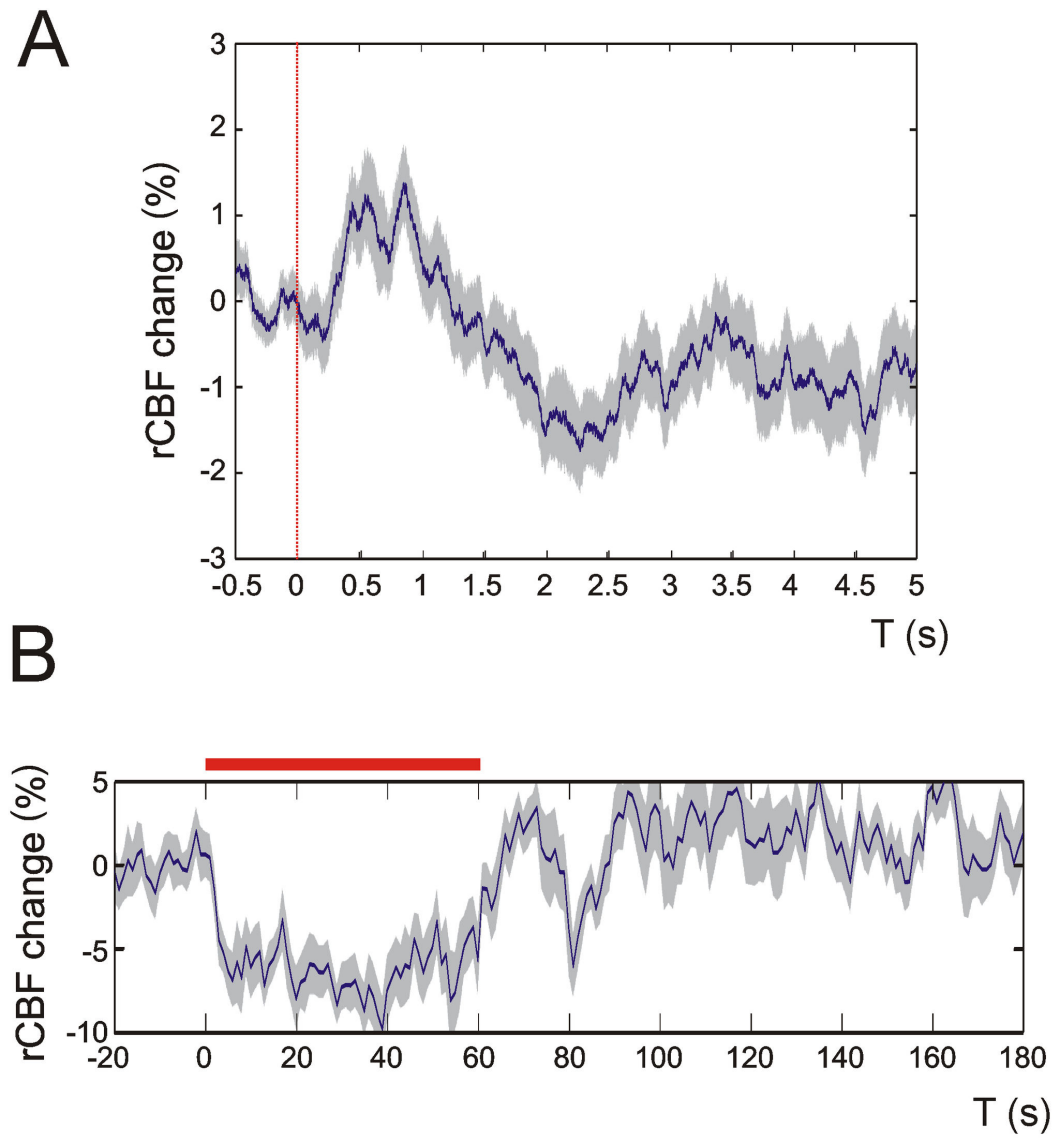
rCBF changes upon multi-whisker stimulation in intact skull recordings. rCBF recordings in intact skull preparations indicate that the craniotomy in our experimental setup did not affect rCBF measurements. Panel **A** shows an average 0.1 Hz recording trace from 100 stimulation events of a P7 mouse. In **B** an average 4 Hz recording is displayed. Note that both traces display similar shapes and characteristics compared with rCBF traces obtained in craniotomy (see Figures 3 and 6). Dashed red line in A: single multi-whisker stimulus, red bar in B: 4 Hz multi-whisker stimulation. Grey shades indicate + SEM. Representative recording traces from 3 P7 animals are displayed.

To verify the validity of recordings rCBF was also measured in intact skull recordings at P7 where we found similar rCBF responses compared with the open cranial window preparation indicating that craniotomy had no impact on neurovascular coupling (Figure **2**). To exclude movement artifacts and to strengthen the sensitivity of recordings we performed a number of controls. Whiskers were stimulated ipsilateral and far away (here the stimulator had no contact with whiskers) and rCBF was recorded in intact skull preparations as mentioned before. Figure **S1** shows that rCBF and MU activity were not affected in ipsilateral and remote control recordings. Here, representative 4 Hz stimulation controls from P30 mice are shown. Figure **S2** demonstrates that in representative 0.1 Hz recording traces breathing rates in P7 and P30 were stable and rhythmic indicating a stable level of urethane anesthesia. During experimental manipulation respirations per minute (RPM) were 193 + 8 RPM at P7 and 169 + 20 RPM at P30. Breathing was found to be rhythmic and stable, no apneic phases were observed during the course of the experiments.

Local field potentials (LFPs) were simultaneously recorded in all experiments and confirmed neural activation as detected by MUA. 

### Immunohistochemistry

In order to study relative myelin basic protein (MBP) expression at P7 and P30 we performed MBP and anti-SMI-31R (axonal marker) co-stainings in cortices of P7 and P30 mice. For immunohistochemical processing brains were removed and snap frozen in cryo glue (Slee Medical GmbH, Germany). Subsequently 20 µm thick coronal slices were prepared using a cryostat (MTC, Slee Medical GmbH, Germany) and fixed in acetone for about 10 minutes. Probes were stored at -20°C until further processing. For immunostainings probes were washed with 0.01 mol/l PBS. Then tissue was blocked and permeabilized with 7% normal donkey serum (Dianova) containing 0.8 % triton in 0.01 PBS for 2 hours at room temperature (RT). After another washing step with PBS 0.01 mol/l probes were incubated with anti-mouse FAB fragment (1:20 in PBS, Jackson Dianova) for 2 hours at RT and washed with PBS 0.01 mol/l. Primary antibodies were incubated in 2 % bovine serum albumin with 0.05 % acid and 0.3 % triton in PBS 0.01 mol/l over night at RT. Primary antibodies were mouse anti-SMI-31R (1:10000, Covance), rat anti-MBP (1:50, Abd Serotec). Following washing secondary antibodies and DAPI (0.5 µg/ml, Sigma) were incubated for 2 hours at RT in 2 % bovine serum albumin, 0.05 % acid. Secondary antibodies were Cy3-conjugated donkey anti-mouse (1:200, Jackson Dianova) and anti-rat donkey DyLight488 (1:200, Bethyl Lab). After a final washing step probes were embedded in Fluoromount.

### Immunohistochemical MBP quantification

For determination of relative MBP expression in P7 and P30 cortices 4 randomly chosen fields per view in cortical layers 1-6 were chosen per animal. Three animals per group were used. Fiji is just ImageJ (NIH software) was used to measure average intensities of randomly chosen fields of view. Average background intensity was substracted from each probe. Images were acquired using a Leica SP5 confocal microscope. Z-stacks at 700 nm thickness were acquired and maximum Z-stack projections were used for analyses. Excitation wavelengths were 405, 488 and 561 nm.

### Data analysis and statistics

LFP data were imported and analyzed offline using MATLAB software version 7.7 (MathWorks). MUA was detected in 200 Hz high-pass filtered signals by applying a threshold at 7 times the baseline SD. The post-stimulus time histograms (PSTHs) were computed by summing up the activity in 100 trials for 0.1 Hz stimulation paradigm (1 ms bin size) and 8 to 11 trials for 4 Hz stimulation paradigm (1 s bin size) and normalized into number of multi-unit spikes per second. All data are expressed as mean + standard error of the mean (SEM). The null hypothesis was rejected at alpha < 0.05. All datasets were analyzed for normalization using the D’Agostino and Pearson method[26]. Correlation was tested for levels of significance using the Spearman test (rCBF/MUA P7 nonparametric dataset) and Pearson test (rCBF/MUA P30 parametric dataset). Data were analyzed with Graphpad 4.02 for windows. The program BiAS (version 9.17 for windows) was used to verify that significantly different findings in the presented report were based on proper sample size. 

## Results

### Neurovascular response to single stimulus multi-whisker stimulation

**Figure 3:**
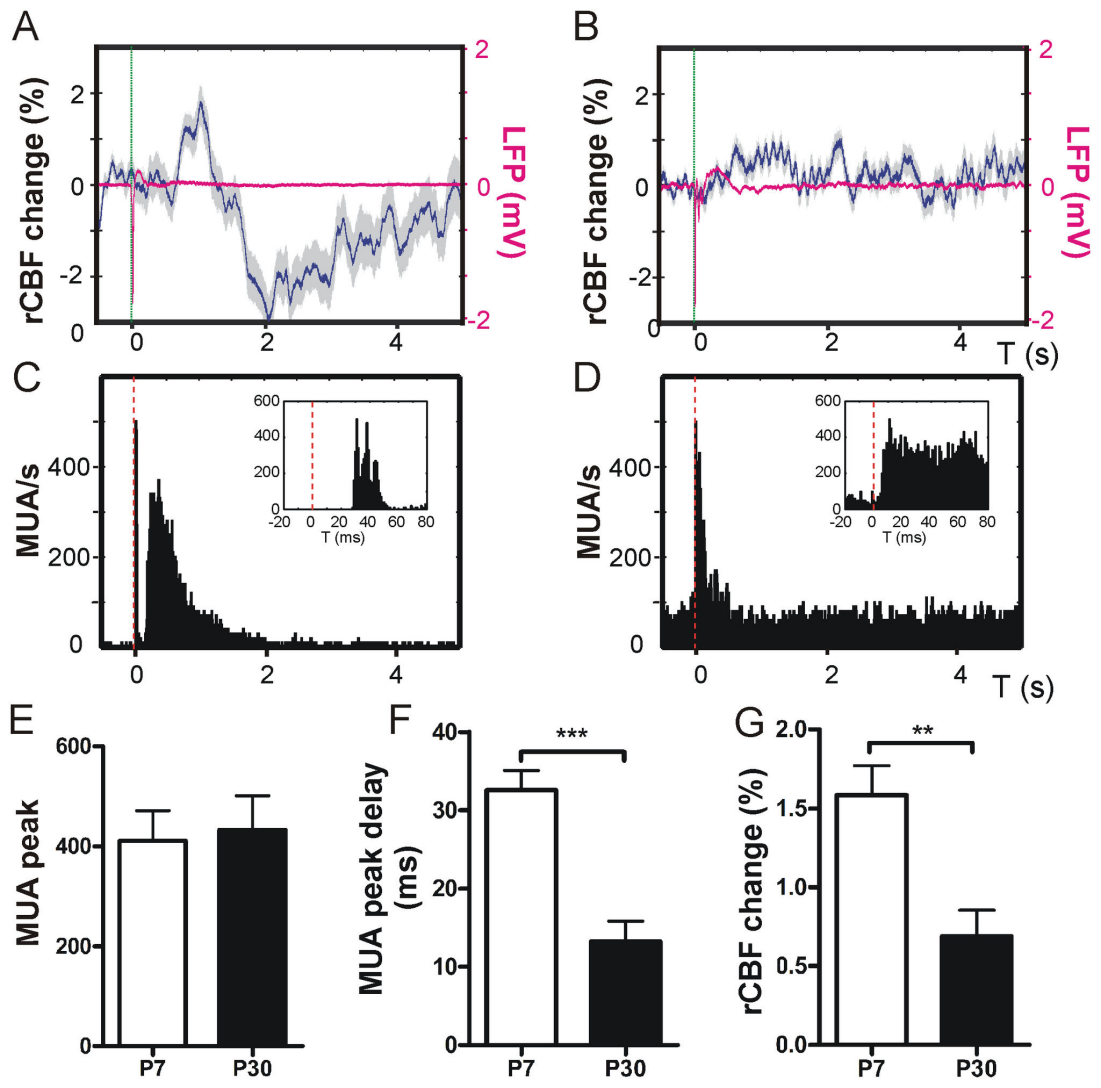
Single multi-whisker stimulation. Single stimulation of multiple whiskers (stimulus is indicated by the dashed green line) resulted in a biphasic rCBF response at P7 with an initial rise in rCBF that was followed by a rCBF decline. The rCBF showed a trend to recover to baseline (representative rCBF trace of 100 averaged 0.1 Hz recordings in one P7 mouse and the corresponding LFP trace in magenta, **A**). At P30 (**B**) single-whisker stimulation evoked a small rise in rCBF (corresponding LFP trace is depicted in magenta). Simultaneous recordings of MUA revealed an initial maximum following stimulation (**C**, representative MUA PSTH plot of 100 recordings in one P7 mouse, inset exemplifies MUA peak delay upon stimulation) that was followed by a delayed MUA response in P7 lasting about 2 seconds. At P30 stimulation resulted in a similar MUA response but the delayed MU activity was not as strong as in P7 mice (representative MUA recording of one P30 mouse **D**). Single multi-whisker stimulation resulted in a similar amount of maximum MUA recruitment at both age groups (**E**). MU activity peak delay was significantly longer at P7 compared with P30 (**F**) whilst the maximum increase of rCBF was higher at P7 compared with P30 within the first 5 seconds after single stimulation (**G**). Grey shades in panels A and B indicate + SEM. Note that SEM does not visualize in LFP traces due to very small SEM.

First, the neurovascular response to a deflection of multiple whiskers at 0.1 Hz was recorded. In P7 we found a biphasic response in rCBF in the barrel cortex. An initial rise in rCBF at 988 + 143 ms peak post-stimulus on average (Figure **3 A**, also note the corresponding LFP trace) was followed by a rCBF decline at about 2 s after stimulation with a subsequent trend to recover. At P30 we were not able to detect this biphasic pattern in rCBF change. Here rCBF increased slightly and returned to baseline (Figure **3 B**, please note the corresponding LFP trace). These changes in rCBF were accompanied by changes in MUA during stimulation in P7 (Figure **3 C**) and P30 animals (Figure **3 D**). In P7 animals single multi-whisker stimulation resulted in an increased MUA lasting for approximately 2 s, which was absent in P30. The maximum initial MUA and LFP peak response was not significantly different at both ages in our setting (P7 MUA peak: 411 + 60 vs. P30 MUA peak 433 + 68, P > 0.05; unpaired t-test, Figure **3 E**; P7 LFP peak 1959 + 508 µV vs. P30 LFP peak 1306 + 182 µV, P > 0.05, unpaired t-test, a representative LFP response during 0.1 Hz is shown in Figure **3 A**, n = 5 animals per group). However the delay in MUA and maximum LFP response upon mechanical whisker deflection was significantly shorter in P30 compared with P7 (MUA max post-stimulus delay P7 32.6 + 2.5 ms vs. P30 13.2 + 2.6 ms, P < 0.001, unpaired t-test, Figure **3 F**; LFP max delay in P7 37.9 + 1.8 ms vs. in P30 26 + 3.4 ms, P < 0.05, unpaired t-test, n = 5 animals, not shown in graph). The overall rCBF increase was significantly higher at P7 compared with P30 (rCBF increase P7 1.6 + 0.2 % vs. P30 0.7 + 0.2 %, P < 0.01, unpaired t-test, n = 5 animals, Figure **3 G**). 

### Expression of cortical MBP is significantly lower at P7 compared with P30

**Figure 4:**
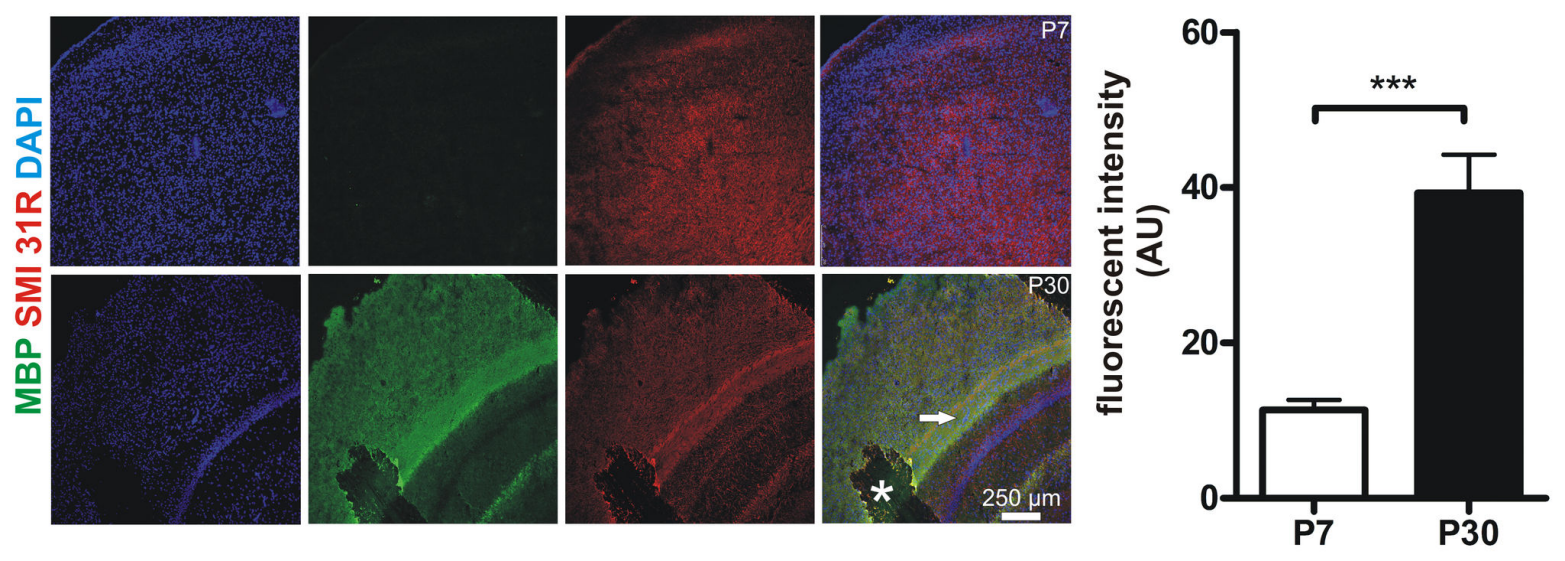
Murine cortices are less myelinated at P7 compared with P30. In order to determine myelination MBP expression was studied in cortices of P7 and P30 mice. Representative z-stack maximum projections (700 nm thickness of stacks) from 3 animals per group are shown (MBP green, the axonal marker SMI 31-R in red, DAPI blue). Note that MBP is nearly absent in the P7 cortex whilst there is high MBP expression at P30. Note the highly myelinated intermediate zone marked with an arrowhead. Asterisk marks a preparation artifact. Quantitative analyses of MBP fluorescence (AU: arbitrary units) demonstrate a significantly smaller MBP expression at P7. Pictures were acquired using equal time exposures and laser intensities.

Because cortical MUA response at P7 was significantly delayed compared with P30 the underlying cellular mechanism were studied in more detail. Nerve conducting velocity is crucially dependent on myelination of nerve fibers[27]. It is known that murine myelination is not fully established at neonatal age[28]. Therefore we analyzed myelination in P7 and P30 mice cortices via immunohistochemical visualization of myelin basic protein (MBP), a key component for proper myelination[28,29]. Here we found a significant difference in MBP levels in P7 compared to P30 animals (cortical MBP fluorescent intensity at P7 11.4 + 1.3 arbitrary units (AU) vs. at P30 39.3 + 4.9 AU, P < 0.0001, unpaired t-test, n = 12 cortical regions of interest from 3 animals per group, Figure **4**). 

### Neuronal excitability decreases upon single whisker stimulation in P7 mice

**Figure 5:**
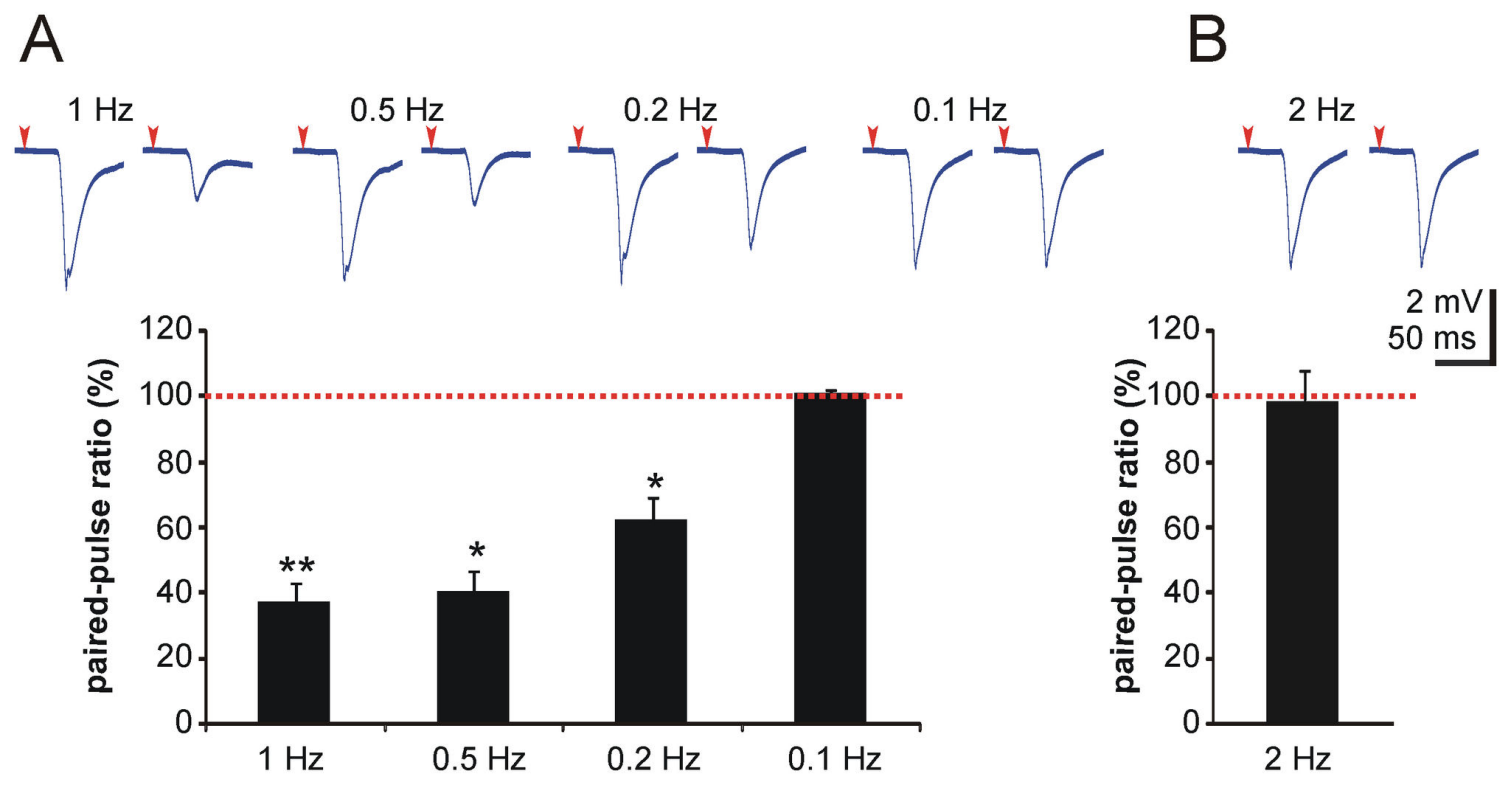
Neuronal excitability following a single whisker stimulation is reduced at P7. In paired pulse experiments neuronal excitability upon single whisker stimulation was assessed for up to 10 seconds after initial stimulus. Paired pulses at 1 Hz to 0.2 Hz revealed a significant reduction in LFP amplitude as shown by representative LFPs in upper traces and bar diagrams at P7 (**A**). Ten seconds after initial stimulus neuronal excitability was fully recovered in P7 mice (**A**). In P30 mice neuronal excitability was restored within 500 ms (Panel **B**). Each paired pulse was applied at an interval of 20 seconds. Red arrowheads: stimulus; ** P < 0.01; * P < 0.05.

As the biphasic response in rCBF change during the first 5 s following single multi-whisker stimulation was found to be highly consistent in every recording in P7 mice, we further investigated this time period regarding neuronal excitability by performing paired pulse experiments on single whiskers. We found that 1 s after initial stimulation the relative neuronal response was significantly reduced as demonstrated by reduced LFP amplitudes compared with initial whisker deflection (1 Hz: 37.6 + 5.6 %, P < 0.01; 0.5 Hz: 40.6 + 6.1 %, P < 0.05; 0.2 Hz: 62.7 + 6.5 %, P < 0.05; 0.1 Hz: 101 + 1.3 %, P > 0.05, n = 3 animals per group, paired t-tests were used, Figure **5 A**). This decrease in LFP amplitude was detectable for up to 5 s of paired pulse intervals whilst after 10 s post-stimulus the neuronal LFP response fully recovered. In P30 mice neuronal excitability already fully recovered within 500 ms post-stimulus (paired pulse at 2 Hz: LFP 98.8 + 8.9 %, P > 0.05, n = 3 animals, paired t-test, Figure **5 B**).

### Changes in rCBF and MUA during prolonged multi-whisker stimulation

**Figure 6:**
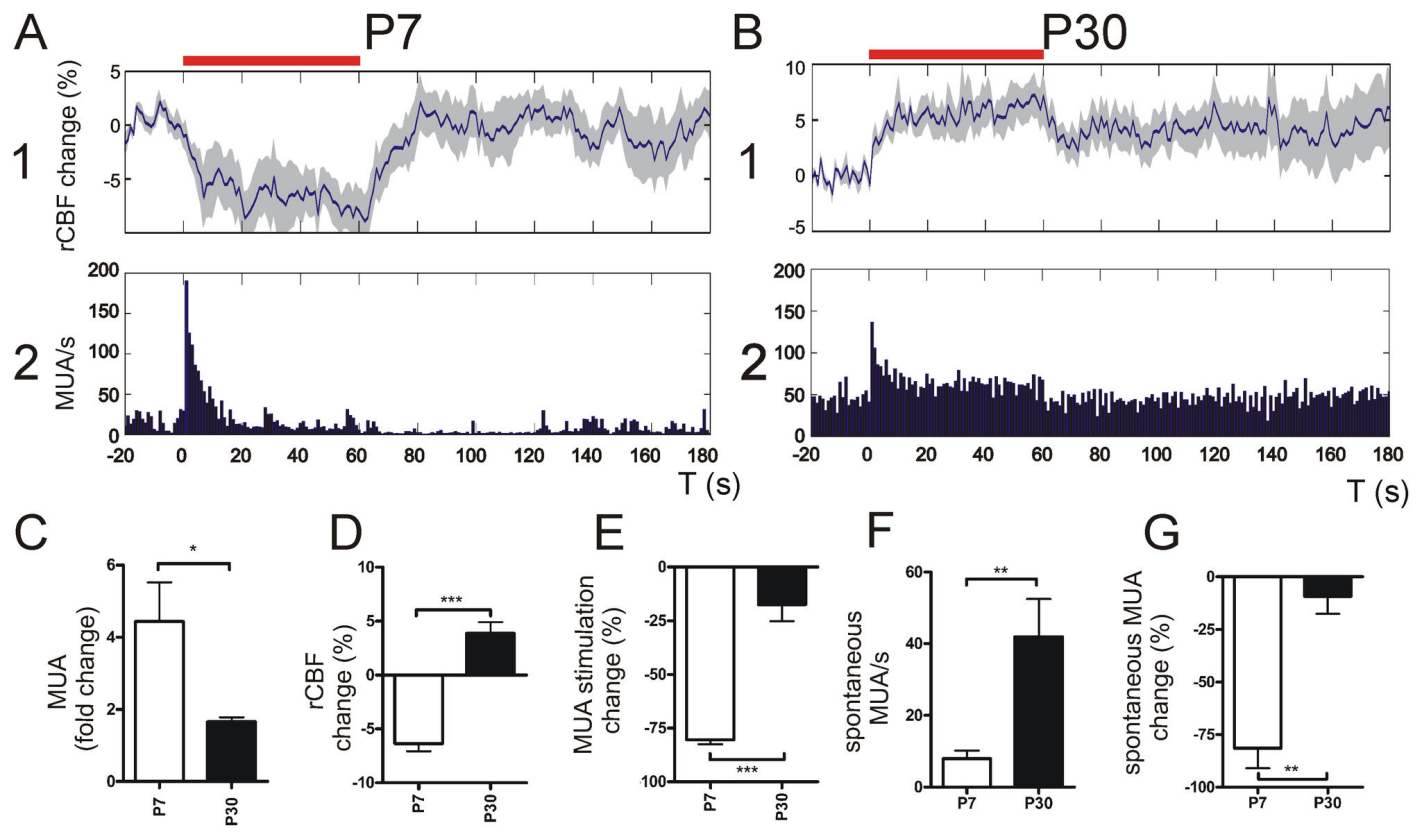
Prolonged multi-whisker stimulation. At P7 prolonged multi-whisker stimulation at 4 Hz resulted in a decline in rCBF. rCBF declined until it reached a plateau and slowly recovered to baseline within 20 - 30 s after the end of stimulation (A 1). In P30 4 Hz stimulation caused a rCBF increase until a plateau was reached and declined after stimulation. In many cases rCBF did not reach baseline levels but remained slightly elevated (B 1). Simultaneous MUA recordings in the barrel cortex of P7 mice demonstrated an initial rise in MUA that decreased during ongoing stimulation (A 2). Note that the baseline MUA after stimulation appears to be reduced compared with MUA before stimulation indicating spontaneous MUAF. At P30 MUA responded in a similar manner as in P7 but the prominent decrease in spontaneous MUA after stimulation was not apparent (B 2). At P7 the MUA increase related to baseline MUA before stimulation was significantly higher than at P30 (**C**). The average rCBF change during stimulation compared with baseline was also significantly different in both age groups. At P7 rCBF decreased while during stimulation rCBF increased at P30 (**D**). MUA decrease over stimulation time was significantly higher in P7 compared with P30 (**E**). In contrast spontaneous MUA before stimulation was higher at P30 compared with P7 (**F**). Statistical analyses of MUA after stimulation compared with MUA levels before stimulation show a significant reduction in spontaneous MUA following 4 Hz whisker deflection in P7 that is much less pronounced in P30 (**G**). Red bar indicates 4 Hz stimulation; *** P < 0.001; ** P < 0.01; * P < 0.05. Grey shades in rCBF panels A and B indicate + SEM.

Under natural conditions, rodents explore their environment and socially interact by intense active whisking[30]. Therefore we performed prolonged whisker stimulation (60 s) at a frequency of 4 Hz. Similar paradigms have already been used in rodent studies analyzing changes in rCBF in the barrel cortex[18–20]. MUA and rCBF changes during 4 Hz stimulation were calculated by relating the average of MUA or rCBF during 60 s of 4 Hz stimulation to the average of MUA or rCBF 10 s before stimulation. Here we found a significant drop in rCBF in P7 mice during stimulation compared with baseline (Figure **6** **A 1**). In contrast in P30 animals rCBF continuously increased during ongoing stimulation until a plateau was reached (**Figure 6 B 1**). After stimulation rCBF returned to baseline in P7 whilst in P30 rCBF generally maintained an elevated level compared to the baseline. At both ages MUA was significantly increased compared to the spontaneous MUA baseline activity (fold MUA change P7: 4.4 + 1.1 vs. P30: 1.7 + 0.1, P < 0.05, unpaired t-test, n = 4 animals; Figure **6** **A 2, B 2, C**). These MUA changes were accompanied by significantly altered rCBF changes in both age groups during whisker deflection (rCBF change P7: -6.4 + 0.9 % vs. P30: 3.9 + 1 %, P < 0.001, unpaired t-test, n = 4 animals; Figure **6 D**). To evaluate MUA changes during stimulation average MUA of the last 10 s during stimulation was related to the first 10 s during stimulation, the value 1 was subtracted in order to calculate the decrease in MUA, changes in % are presented. Here MUA decreased in the course of stimulation at both ages but this effect was significantly higher in P7 than in P30 animals (MUA decrease during stimulation P7: -80.2 + 2.6 % vs. P30: -17.5 + 7.6 %, P < 0.001, unpaired t-test, n = 4 animals; Figure **6 E**). As visualized in the MUA graphs spontaneous MUA activity levels in P7 pups were significantly smaller than in P30 animals. The average spontaneous MUA in a 10 s period before stimulation also differed between both age groups (spontaneous MUA P7 9.7 + 1.9 vs. P30 41.9 + 10.5, P < 0.05, unpaired t-test, n = 4 animals, Figure **6 F**). To elucidate if prolonged multi-whisker deflection had an impact on spontaneous MUA following stimulation the average spontaneous MUA of a 10 s period directly after stimulation was related to the average spontaneous MUA within 10 s ahead of 4 Hz stimulation. Spontaneous MUA after prolonged multi-whisker deflection in P7 mice was suppressed significantly stronger compared with P30 (spontaneous MUA change P7: -81.5 + 9.5 % vs. P30: -9.4 + 8.1 %, P < 0.01, unpaired t-test, n = 4 animals, Figure **6 G**). 

**Figure 7:**
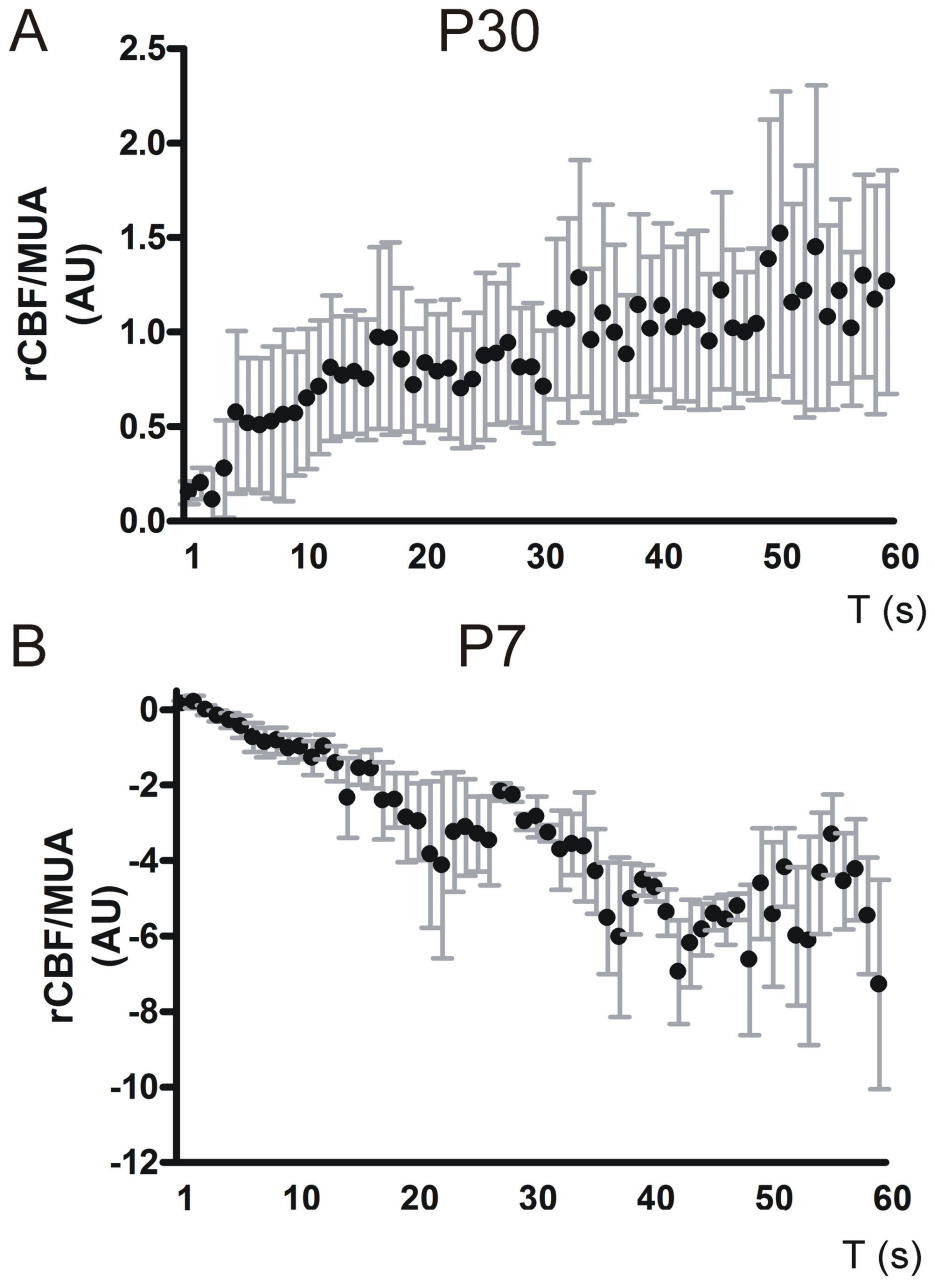
Correlation of rCBF/MUA ratio and stimulation time. rCBF values were correlated to corresponding MUA and were plotted against stimulation time in arbitrary units (AU). rCBF per MUA increases over stimulation in P30 mice and is significantly positively correlated with stimulation duration (**A**). In contrast rCBF decreased per MUA over stimulation time in P7 mice. Here the correlation coefficient was negatively correlated (**B**). Error bars indicate + SEM.

Due to the observation of a highly prominent discrepancy in rCBF change with ongoing stimulation at 4 Hz we correlated rCBF changes to MUA activity at corresponding time points. Here we found a significant positive correlation in rCBF increase related to MUA over stimulation time in P30 mice (Pearson r = 0.9, P < 0.0001, n = 4 animals, Figure **7 A**). That was in contrast to a highly significant negative correlation regarding rCBF/MUA ratio during stimulation in P7 mice (Spearman r = -0.9, P < 0.0001, n = 4 animals, Figure **7 B**). Here rCBF per MUA decreased during stimulation. 

## Discussion

Here we describe that NVC in the rodent whisker-to-barrel cortex undergoes a significant shift during early postnatal development. Our findings indicate that cortical sensory perception is already well established in the P7 barrel cortex. At P7 a single multi-whisker deflection activates a similar number of spiking neurons as in P30 mice. This is supported by our observation that the maximum peak number of MUA upon single multi-whisker deflection at 0.1 Hz is similar in P7 and P30 animals. In comparison to MUA, LFPs give information on a rather large cortical area (few millimeters around the electrode[6,31]) that reflect input signals within a neuronal network. Because the maximum amplitude in LFPs did not significantly differ in both age groups we conclude that besides a similar activation of neural cell numbers the synchronized input of these cellular neural networks also seems to be similar[6]. One of the most significant differences at 0.1 Hz multi-whisker stimulation was the delay in LFP and MUA maximum. Based on our finding of relatively low MBP expression in the cortex of P7 mice compared with P30 we suggest that this delay is likely due to a reduced level of myelination at P7, which results in a reduced nerve conduction velocity at that age. This is in good agreement with previous findings documenting that myelination of the mural CNS begins after birth and is not fully established at P7[29,32]. Another important result was the presence of a biphasic rCBF response at P7, which was absent at P30. We speculate that the initial rise in rCBF may serve as a mechanism for stronger activation of the developing cortical network. At P7 the initial MUA peak was followed by prolonged MUA lasting for a couple of seconds which was not the case in P30 mice. Our data indicate that despite a similar maximal MUA peak at P7 and P30 a single stimulus causes a longer lasting MUA in P7 than P30. However, in the P7 cortex neuronal excitability upon a single stimulus is greatly decreased. This is reflected by our paired pulse experiments in which LFPs were reduced for up to 5 seconds and restored at 0.1 Hz intervals. 

Maintaining neuronal processing is an energy consuming process[3,5,33] that is supported by increases in cerebral blood flow. 

At P30 rCBF increases per MUA over stimulation time while it declines at the age of P7. This conclusion is supported by several lines of evidence in our experimental setting. First we found that during multi-whisker stimulation MUA was significantly reduced at P7 compared with P30 in the course of 4 Hz stimulation. Second in P7 animals we detected a significant decrease of spontaneous MUA after prolonged stimulation compared to spontaneous MUA before stimulation that was less evident in P30. We refer to this finding as evoked “multi-unit activity fatigue” (MUAF) in the developing barrel cortex. The results on prolonged multi-whisker stimulation implicate that the immature brain is not capable to maintain prolonged neural processing information on a rather stable level as we found in P30 animals and that this is associated with a decline in rCBF whilst at P30 rCBF increases to reach a plateau. This different response in hemodynamics in the sensory cortex upon sensory stimulation is supported by data by Kozberg et al.[14]. The authors from this study have shown a shift from a negative to a positive cortical hemodynamic response upon electrical hindpaw stimulation in brain development in rats. However Kozberg et al. have not performed electrophysiological recordings and used electrical hindpaw stimulation which is another experimental condition than in our whisker-to-barrel cortex study. 

It is however unclear if different rCBF responses may have an effect on neural activity itself.

Takuwa et al. have recently shown that long-term hypoxia lowers rCBF increase upon whisker air puff stimulation without an effect on neural activity[34]. These results and data from Nielsen and Lauritzen[35] indicate that a lack of rCBF increase does not necessarily affect neural activity. We would like to stress that we have no experimental evidence regarding the mechanisms underlying the age-dependent differences in NVC at the cellular level. However it is known that neurotransmitters e.g. GABA and glutamate can have an effect on cerebral vessel constriction and dilation[5,36,37]. Further it is has been documented that levels of excitation and inhibition undergo developmental changes during maturation in the mammalian CNS[4,38]. Our results on paired pulse experiments are in agreement with these reports and indicate that neuronal excitability decreases for up to 5 seconds following multi-whisker single stimulation at P7 whilst it already recovers after 500 ms at P30. This observation and the detected MUA fatigue indicate that a decreased level of neuronal excitability upon repetitive stimulation is present at P7 compared with P30. Future studies may address if age dependent changes in the release of neurotransmitter e.g. GABA or glutamate may affect NVC and explain the observed discrepancies in NVC in the developing whisker-to-barrel sensory system. 

This finding is somewhat in contrast to some reports the literature in which it is documented that a sensory stimulation leads to an increase of rCBF in the immature brain which has been shown recently in human preterms non-invasely[12]. The authors of that study however measured changes in cerebral blood flow and oxygen consumption whilst neural responses were not recorded. Our findings that are based on invasive MUA, LFP and simultaneous rCBF recordings, rather support recent data obtained with multispectral optical intrinsic imaging regarding a developmental change in local cortical hemodynamics upon sensory stimulation[14]. We suggest that in the developing cortex sensory processing does not necessarily increase rCBF but may also result in rCBF decline and MUAF depending on the stimulation paradigm. 

In addition we would like to stress that the observed MUA change during 4 Hz stimulation at P7 is somewhat remarkable: Compared with baseline MUA increases up to 4-5 times in the developing barrel cortex, in the adolescent we found only a 2-3 fold increase. This indicates that the developing barrel cortex is able to maintain MUA about twice as high as the adolescent cortex compared with baseline levels, which we interpret as an enormous performance of the developing cortex compared with the adolescent cortex. However, this finally results in MUAF at the level of the developing cortical column. Further we would like to emphasize that a limitation of our study is that recordings were obtained in anesthetized animals and that anesthesia may affect NVC studies. We chose the anesthetic agent urethane which has been shown to have a balanced effect on neurotransmitters and is therefore regarded as a suitable anesthetic agent for NVC analyses[20]. However our experimental setup has an important advantage: we specifically chose the whisker-to-barrel cortex system because here it is possible to induce sensory perception in a physiological non-painful way by deflecting whiskers[39]. Other approaches to evoke sensory responses in the cortex e.g. electrical stimulation have been demonstrated to induce changes in systemic blood pressure[14] that may alter cerebral perfusion in neonates which may be linked to the induction of a stress response due to the electrical stimulus and insertion of an electrode into e.g. rodent paws.

Li and others have shown that changes in rCBF vary depending on the recording depth[35,40] in the rodent cerebral cortex. In our experimental setting we used LDF probes with a penetration depth of about 1 mm[41]. Because the LDF probe was positioned about 300 - 500 µm above the pia mater the obtained LDF signal was mostly derived from upper cortical layers with an approximate depth of 0.5 mm. Neural activity was obtained from electrodes that were inserted into a depth of 200 - 300 µm. Therefore our results mostly reflect NVC at upper cortical layers. We would like to point out this fact as NVC may be different in deeper cortical layers[35,40].

As documented above more work is needed to unravel the mechanisms that mediate the observed developmental switch in neurovascular coupling in the barrel cortex. Based on our results we suggest that NVC in the sensory whisker-to-barrel neocortex undergoes a significant switch during cortical development which may be of key relevance to establish long term and robust sensory processing. Several studies investigating NVC have described increases of 20-30 % of rCBF in the barrel cortex upon whisker stimulation[18,34]. However many of these studies were performed in rodents at 6-8 weeks of age[18,19,35,42]. In the current study we investigated NVC in 1 week and about 4 week old animals which are not fully mature. The changes in rCBF that we detected were not higher than 10 %. However in line with our data rCBF changes fewer than 10 % have been reported[12] in human preterm babies. 

## Conclusion

Our data demonstrate that NVC in the whisker-to-barrel cortex undergoes significant changes during postnatal brain maturation. The underlying mechanisms remain to be determined. However we suggest that our findings may be of importance to correctly interpret and analyze neurovascular coupling and brain function in the developing brain.

## Supporting Information

Figure S1 **Neurovascular control recordings.** In ipsilateral stimulations of multiple whiskers at 4 Hz for 60 seconds a response in rCBF and MUA (Panel **A**) became not apparent. During remote stimulations at 4 Hz rCBF and MUA were also not affected (**B**). One representative recording from 3 P30 animals per group are depicted. Shades of grey in rCBF traces indicate + SEM.
(TIF)

Figure S2 **Simultaneous recording of breathing cycles, LFPs, MUA and rCBF.** Several parameters were acquired simultaneously throughout the experiments. Line 1 indicates breathing rhythms detected by a piezoelectric element as previously described[24] displaying stable and rhythmic respiration; line 2 displays the stimulus application (here 0.1 Hz) whilst in 3 LFPs and 4 MUA are displayed; line 5 depicts changes in rCBF. Recordings from a P7 mouse are shown in panel **A**. Traces in **B** show recordings from a P30 mouse. Note that before stimulation there is no spontaneous LFP change in P7 (**A**, line 3) compared with P30 (**B**, line 3). Changes in MUA are accompanied by alterations in LFPs (lines 3 and 4, A, B). A single stimulus resulted in a stronger and longer lasting MUA response in P7 as compared to P30. Note that simultaneous recordings from single trials without averaging are displayed which explains the absence of + SEM and why the LDF traces are not as smooth as in other recordings displayed in this report.
(TIF)
