# Supplementary figures and images for: Developmental Switch in Neurovascular Coupling in the Immature Rodent Barrel Cortex
Source: PLoS One. 2013 Nov 5;8(11):e80749. doi: 10.1371/journal.pone.0080749 (PMC3818260; doi:10.1371/journal.pone.0080749)

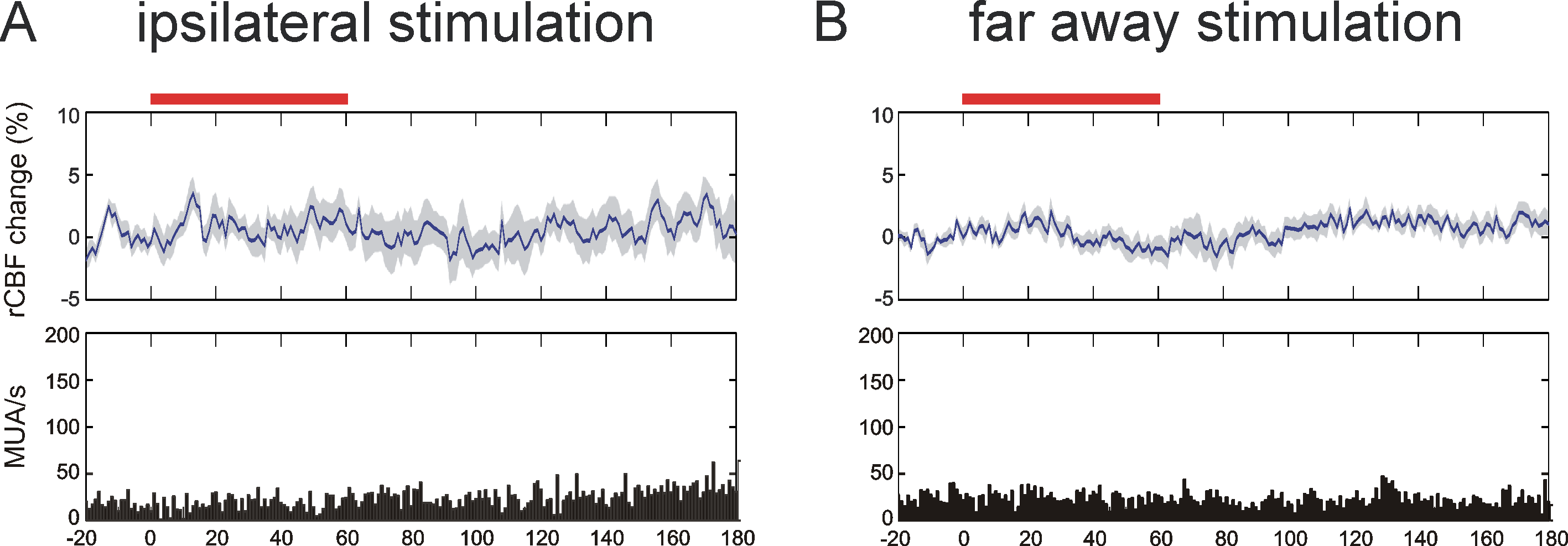

Supplement: Figure S1 — Neurovascular control recordings. In ipsilateral stimulations of multiple whiskers at 4 Hz for 60 seconds a response in rCBF and MUA (Panel A) became not apparent. During remote stimulations at 4 Hz rCBF and MUA were also not affected (B). One representative recording from 3 P30 animals per group are depicted. Shades of grey in rCBF traces indicate + SEM. (TIF) [file pone.0080749.s001.tif]

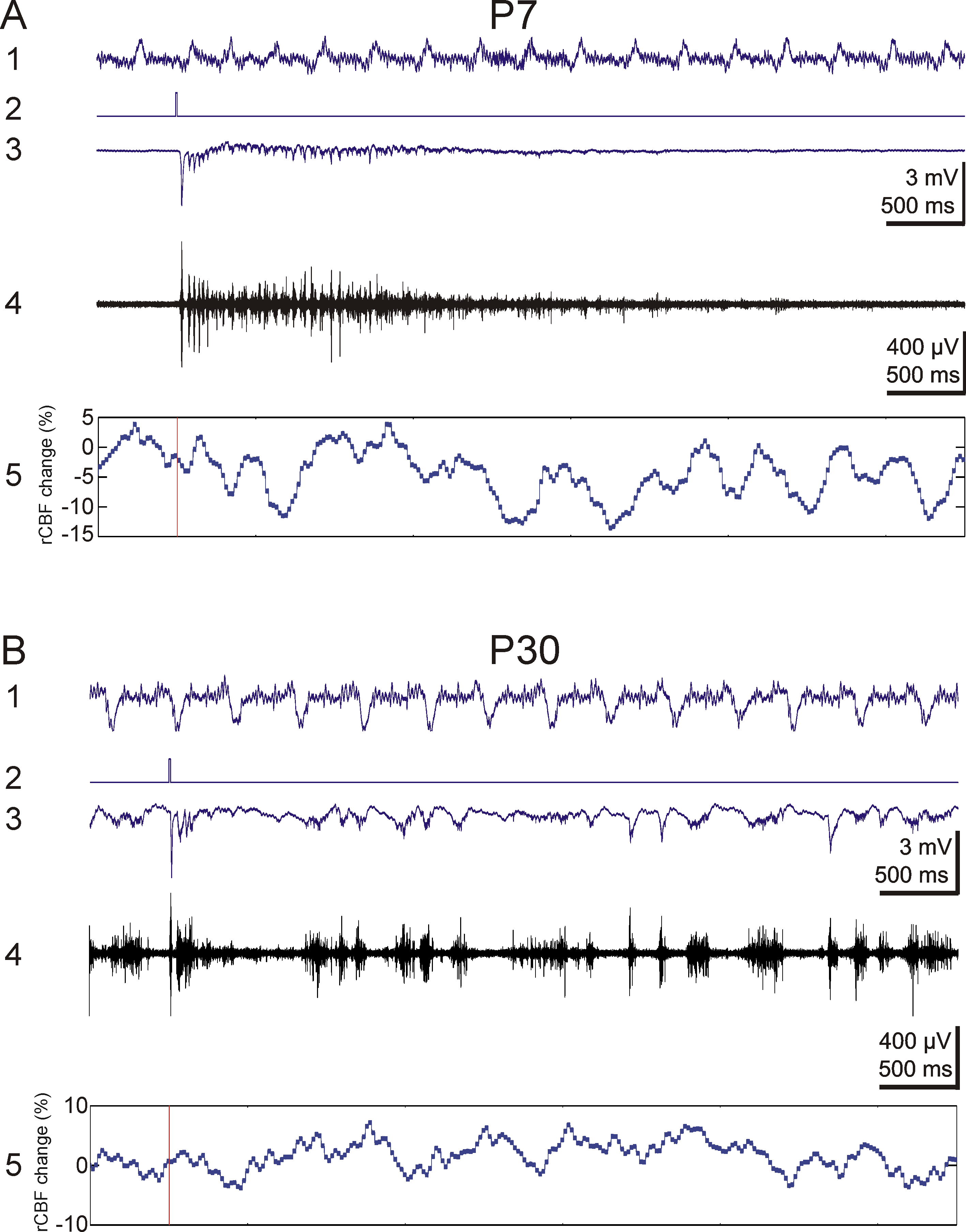

Supplement: Figure S2 — Simultaneous recording of breathing cycles, LFPs, MUA and rCBF. Several parameters were acquired simultaneously throughout the experiments. Line 1 indicates breathing rhythms detected by a piezoelectric element as previously described[24] displaying stable and rhythmic respiration; line 2 displays the stimulus application (here 0.1 Hz) whilst in 3 LFPs and 4 MUA are displayed; line 5 depicts changes in rCBF. Recordings from a P7 mouse are shown in panel A. Traces in B show recordings from a P30 mouse. Note that before stimulation there is no spontaneous LFP change in P7 (A, line 3) compared with P30 (B, line 3). Changes in MUA are accompanied by alterations in LFPs (lines 3 and 4, A, B). A single stimulus resulted in a stronger and longer lasting MUA response in P7 as compared to P30. Note that simultaneous recordings from single trials without averaging are displayed which explains the absence of + SEM and why the LDF traces are not as smooth as in other recordings displayed in this report. (TIF) [file pone.0080749.s002.tif]
